# Multisensory Concept Learning Framework Based on Spiking Neural Networks

**DOI:** 10.3389/fnsys.2022.845177

**Published:** 2022-05-12

**Authors:** Yuwei Wang, Yi Zeng

**Affiliations:** ^1^Research Center for Brain-inspired Intelligence, Institute of Automation, Chinese Academy of Sciences, Beijing, China; ^2^School of Artificial Intelligence, University of Chinese Academy of Sciences, Beijing, China; ^3^Center for Excellence in Brain Science and Intelligence Technology, Chinese Academy of Sciences, Shanghai, China; ^4^National Laboratory of Pattern Recognition, Institute of Automation, Chinese Academy of Sciences, Beijing, China

**Keywords:** concept learning, multisensory, spiking neural networks, brain-inspired, Independent Merge, Associate Merge

## Abstract

Concept learning highly depends on multisensory integration. In this study, we propose a multisensory concept learning framework based on brain-inspired spiking neural networks to create integrated vectors relying on the concept's perceptual strength of auditory, gustatory, haptic, olfactory, and visual. With different assumptions, two paradigms: Independent Merge (IM) and Associate Merge (AM) are designed in the framework. For testing, we employed eight distinct neural models and three multisensory representation datasets. The experiments show that integrated vectors are closer to human beings than the non-integrated ones. Furthermore, we systematically analyze the similarities and differences between IM and AM paradigms and validate the generality of our framework.

## 1. Introduction

Concept learning, or the ability to recognize commonalities and accentuate contrasts across a group of linked events in order to generate structured knowledge, is a crucial component of cognition (Roshan et al., 2001). Multisensory integration benefits concept learning (Shams and Seitz, 2008) and plays an important role in semantic processing (Xu et al., 2017; Wang et al., 2020). For example, when we learn the concept of “tea,” acoustically, we will perceive the sound of pouring water and brewing, the sound of clashing porcelain, the sound of drinking tea; on taste, we can feel the tea is a bit bitter, astringent or sweet; in touch, tea is liquid and we can feel its temperature; on smell, we can perceive the faint scent and visually, it often appears together with the teapot or tea bowl, and the tea leaves will have different colors. Combining information from multiple senses can produce enhanced perception and learning, faster response times, and improved detection, discrimination, and recognition capabilities (Calvert and Thesen, 2004). In the brain, multisensory integration occurs mostly in the superior colliculus according to existing studies (Calvert and Thesen, 2004; Cappe et al., 2009). Multisensory integration is a field that has attracted the interest of cognitive psychologists, biologists, computational neuroscientists, and artificial intelligence researchers. The term “multisensory concept learning” is used in this work to describe the process of learning concepts using a model that mimics humans and combines information from multiple senses.

For the computational models of multisensory integration, cognitive psychologists' models are usually focused on model design and validation from the mechanism of multisensory integration. These models are highly interpretable, taking neuroimaging and behavioral studies into consideration. The cue combination model based on Bayesian decision theory is a classical model for analyzing multisensory integration in cognitive psychology. It mainly models the stimuli of different modalities as the likelihood functions of Gaussian (Ursino et al., 2009, 2014) or Poisson (Anastasio et al., 2014) distributions with different parameters, and calculates the best combination of each modality that makes the maximum posterior distribution through the assumption of conditional independence and Bayesian rules. Anastasio et al. built a model of visual and auditory fusion that combines neuronal dynamic equations with feedback information, and this model verified that multimodal stimuli have less response time than unimodal stimuli (Anastasio et al., 2014). Parise et al. proposed multisensory correlation detector based models to describe correlation, lag, and synchrony across the senses (Parise and Ernst, 2016). A purely visual haptic prediction model is presented by Gao et al. (2016) with CNNs and LSTMs, which enables robots to “feel” without physical interaction. Gepner et al. (2015) developed a linear-nonlinear-Poisson cascade model that incorporates information from olfaction and vision to mimic Drosophila larvae navigation decisions, and the model was able to predict Drosophila larvae reaction to new stimulus patterns well.

For artificial intelligence researchers, they have proposed different types of multisensory integration models based on the available data and machine learning methods, such as direct concatenation (Kiela and Bottou, 2014; Collell et al., 2017; Wang et al., 2018b), canonical correlation analysis (Silberer et al., 2013; Hill et al., 2014), singular value decomposition of the integration matrix (Bruni et al., 2014), multisensory context (Hill and Korhonen, 2014), autoencoders (Silberer and Lapata, 2014; Wang et al., 2018a), and tensor fusion networks (Zadeh et al., 2017; Liu et al., 2018; Verma et al., 2019). These works are mostly focused on concept learning and sentiment analysis tasks and are based on modeling of speech, text, and image data, which are commonly utilized in AI.

To our knowledge, no work exists to model the five senses of vision, hearing, touch, taste, and smell together. This might be because controlling elements for experimental design is challenging for cognitive psychologists, while data for some modalities is difficult to get using perceptrons for AI researchers. Meanwhile, cognitive psychologists have published several multisensory datasets by asking volunteers how much they perceive a specific concept through their auditory, gustatory, tactile, olfactory, and visual senses in order to establish the strength of each modality. This provides a solid basis for the design of a multisensory integration model that includes these five modalities. In this article, we will model multisensory integration using brain-like spiking neural networks and merge input from five different modalities to generate integrated representations.

This paper is organized as follows: Section 2 will introduce relevant studies to our model, such as multisensory datasets and fundamental SNN models; Section 3 will describe the multisensory concept learning framework based on SNNs, which includes the Independent Merge and Associate Merge paradigms. Section 4 will exhibit the experiments, and the final section will explore the future works.

## 2. Related Works

### 2.1. Multisensory Concept Representation Datasets

**Figure 1:**
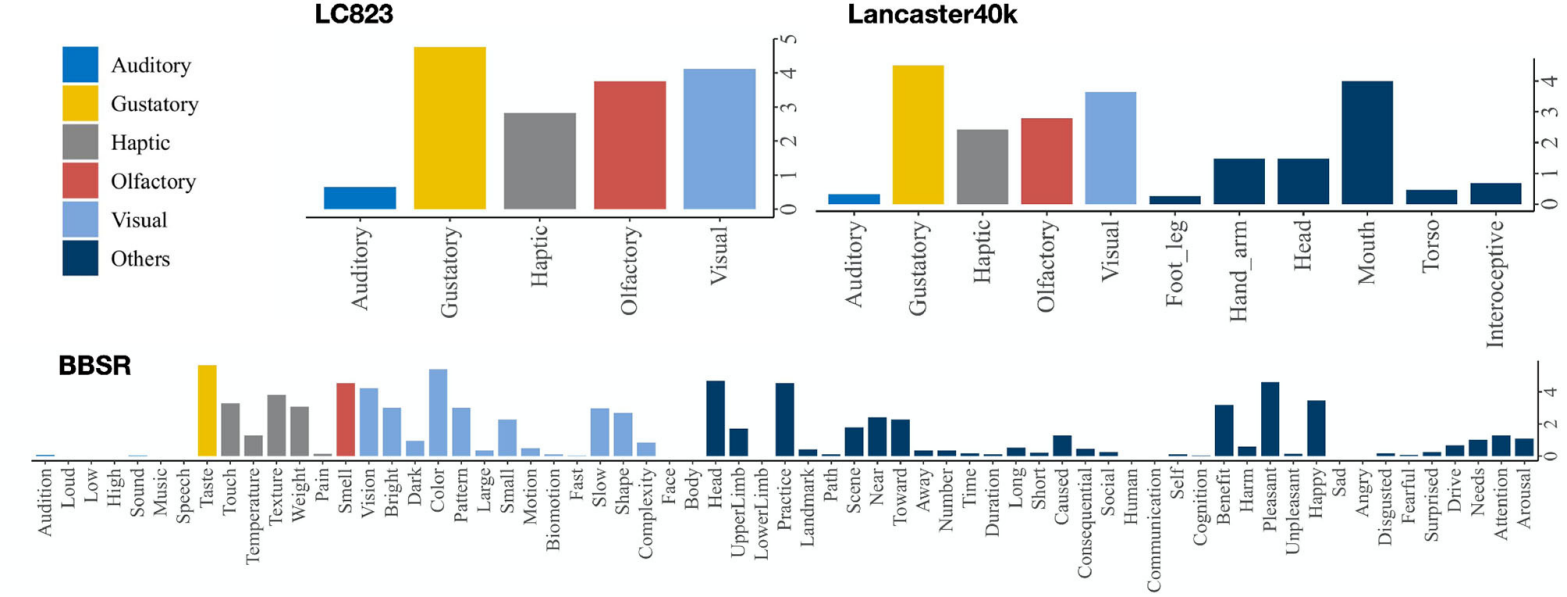
The concept “honey” in multisensory datasets.

Cognitive psychologists label the multisensory datasets of concepts by asking volunteers how much each concept is acquired through a specific modality and introducing statistical methods to establish the representation vector for each concept. The pioneering work in this area is by Lynott and Connell (2013), who proposed modality exclusivity norms for 423 adjective concepts (Lynott and Connell, 2009) and 400 nominal concepts on strength of association with each of the five primary sensory modalities (auditory, gustatory, haptic, olfactory, visual). In this article, we combine these two datasets of their previous works and denote them as LC823. Lancaster Sensorimotor Norms were published by Lynott et al. (2019), which included six perceptual modalities (auditory, gustatory, haptic, interoceptive, olfactory, visual) and five action effectors (foot/leg, hand/arm, head, mouth, torso). This dataset (we denote as Lancaster40k) is the largest ever, with 39,707 psycholinguistic concepts (Lynott et al., 2019). Binder et al. (2016) constructed a set of brain-based componential semantic representation (BBSR) with 65 experienced attributes, including sensory, motor, spatial, temporal, affective, social, and cognitive experiences, relying on more recent neurobiological findings. This dataset contains 535 concepts and does an excellent work of separating a priori conceptual categories and capturing semantic similarity (Binder et al., 2016). Figure 1 shows the the concept “honey” in the multisensory concept representation datasets mentioned.

We'll concentrate on the effect of five forms of senses in this article: vision, touch, sound, smell, and taste. In BBSR, we employ the average value of the sub-dimensions corresponding to these five senses, while using the first five dimensions of Lancaster40k.

### 2.2. Basic Neuron and Synapse Models

Spiking neural networks (SNNs) are commonly referred to be the third generation of neural network models since theyareinspired by current discoveries in neuroscience (Maass, 1997). Neurons are the basic processing units of the brain. They communicate with each other *via* synapses. When the membrane potential reaches a threshold, a spike is produced. External stimuli are conveyed by firing rate and the temporal pattern of spike trains (Rieke et al., 1999; Gerstner and Kistler, 2002). SNNs integrate temporal information into the model and are capable of accurately describing spike timing with dynamic changes in synaptic weights which are more biologically plausible. We will use SNNs as the foundation of our model to build a human-like multisensory integration concept learning framework. Here, we briefly outline the neural and synaptic models that will be used in this research.

#### 2.2.1. IF Neural Model

The integrate-and-fire (IF) model is a large family of models which assumes that a membrane potential threshold controls the spikes of neurons. A spike is fired when the somatic membrane potential exceeds the threshold, and the membrane potential is resumed to reset potential (Gerstner and Kistler, 2002). The neural processing is properly formalized by the model. In this article, we follow a standard implementation (Troyer and Miller, 1997), and the membrane potential *v*(*t*) obeys


(1) $$\begin{array}{l} \tau_{IF}\frac{dv\left(t\right)}{dt}=v_{rest}-v\left(t\right)+g_{e}\left(t\right)\left(E_{e}-v\left(t\right)\right)\\ \;if\;v\left(t\right)>v_{th},\;v\left(t\right)\leftarrow v_{r} \end{array}$$

with the membrane time constant τ_*IF*_ = 20 *ms*, the resting potential *v*_*rest*_ = −14 *mV*, the threshold for spike firing *v*_*th*_ = 6 *mV*, the reset potential *v*_*r*_ = 0 *mV*, and excitatory potential *E*_*e*_ = 0 *mV*. Synaptic inputs are modeled as conductance *g*_*e*_ changes with $\tau_{e}\frac{dg_{e}}{dt}=-g_{e}$, where τ_*e*_ = 5 *mV*.

#### 2.2.2. LIF Neural Model

The leaky integrate-and-fire (LIF) neuron model is one of the most popular spiking neuron models because it is biologically realistic and computationally easy to study and mimic. The LIF neuron's subthreshold dynamics are described by the equation below:


(2) $$\begin{array}{l} \tau_{LIF}\frac{dv\left(t\right)}{dt}=v_{rest}-v\left(t\right)+R_{m}I\\ \;if\;v\left(t\right)>v_{th},\;v\left(t\right)\leftarrow v_{r} \end{array}$$

In this paper, the membrane resistance constance *R*_*m*_ = 1, τ_*LIF*_ = 20, *v*_*rest*_ = 1.05, *v*_*th*_ = 1, and *v*_*r*_ = 0.

#### 2.2.3. Izhikevich Neural Model

Izhikevich model was first proposed in 2003 to replicate spiking and bursting behavior of known types of cortical neurons. The model combines the biological plausibility of Hodgkin and Huxley (1952) dynamics with the computing efficiency of integrate-and-fire neurons (Izhikevich, 2003). Biophysically accurate Hodgkin-Huxley neural models are reduced to a two-dimensional system of the following dynamics ordinary with bifurcation methods:


(3) $$\begin{array}{l} \frac{dv\left(t\right)}{dt}=0.04v{\left(t\right)}^{2}+5v\left(t\right)+140-u\left(t\right)+I,\\ \;\frac{du}{dt}=a\left(bv\left(t\right)-u\left(t\right)\right)\\ if\;v\left(t\right)>v_{th},\;v\left(t\right)\leftarrow c\;and\;u\left(t\right)\leftarrow u\left(t\right)+d \end{array}$$

where the time scale of the recovery variable *u* is described by the parameter *a*, the sensitivity of the recovery variable *u* to subthreshold changes of the membrane potential *v* is described by the parameter *b*, the parameter *c* defines the membrane potential *v*'s after-spike reset value, which is induced by quick high-threshold *K*^+^ conductances and after-spike reset of the recovery variable *u* induced by slow high-threshold *Na*^+^ and *K*^+^ conductances is described by the parameter *d* (Izhikevich, 2003).

The model simulates the spiking and bursting activity of known kinds of cortical or thalamic neurons such as resonator (RZ), fast spiking (FS), intrinsically bursting (IB), low-threshold spiking (LTS), regular spiking (RS), chattering (CH), and thalamo-cortical (TC) based on these four parameters. These models are employed extensively in our work and details are illustrated in Table 1.

**Table 1:** Izhikevich models.

**Neurons**	**Izhikevich parameters**			
	**a**	**b**	**c**	**d**
RZ (resonator)	0.10	0.25	−65	2
FS (fast spiking)	0.10	0.20	−65	2
IB (intrinsically bursting)	0.02	0.20	−55	4
LTS (low-threshold spiking)	0.02	0.25	−65	2
RS (regular spiking)	0.02	0.20	−65	8
CH (chattering)	0.02	0.20	−50	2
TC (thalamo-cortical)	0.02	0.25	−65	0.05

#### 2.2.4. STDP Synapse Models

Spike-timing-dependent plasticity (STDP) is a biological process that modifies the strength of neural connections in the brain. Learning and information storage in the brain, as well as the growth and refinement of neural circuits throughout brain development, are thought to be influenced by STDP (Bi and Poo, 2001). The typical STDP model is used in this research, and the weight change Δ*w* of a synapse relies on the relative time of presynaptic spike arrivals and postsynaptic spike arrivals. Δ*w* = Σ_*t*_*pre*__Σ_*t*_*post*__*W*(*t*_*post*_ − *t*_*pre*_), where the function *W*(·) is defined as:


(4) $$\begin{array}{l} W\left(\Delta t\right)=\{\begin{array}{c} A_{+}exp\left(\frac{\Delta t}{\tau_{+}}\right)\;\Delta t>0\\ -A_{-}exp\left(-\frac{\Delta t}{\tau_{-}}\right)\;\Delta t<0 \end{array} \end{array}$$

When implement STDP, we follow the way of Brian2 (Stimberg et al., 2019), which defines two variables *a*_*pre*_ and *a*_*post*_ as the “traces” of of pre- and post-synaptic activity, governed by the following differential equations


(5) $$\begin{array}{l} \;\tau_{pre}\frac{a_{pre}}{dt}=-a_{pre}\\ \tau_{post}\frac{a_{post}}{dt}=-a_{post} \end{array}$$

Once a presynaptic spike occurs, the presynaptic trace is updated and the weight is modified according to the rule


(6) $$\begin{array}{l} a_{pre}\leftarrow a_{pre}+A_{pre}\\ \;w\leftarrow w+a_{post} \end{array}$$

And when a postsynaptic spike occurs:


(7) $$\begin{array}{l} a_{post}\leftarrow a_{post}+A_{post}\\ \;w\leftarrow w+a_{pre} \end{array}$$

This is proved to be equivalent for the two kinds of STDP formulations. And, in this article τ_*pre*_ = τ_*post*_ = 1*ms*.

## 3. The Framework of Multisensory Concept Learning Framework Based on Spiking Neural Networks

We present a multisensory concept learning framework based on SNNs in this part. The model's input is a multisensory vector labeled by cognitive psychologists, with an integrated vector as the output following SNNs merging. Since there is no biological study to show whether the information of multiple senses is independent or associated before integration, two different paradigms: Independent Merge (IM) and Associate Merge (AM) are designed in our framework. The types of inputs and outputs are the same for both paradigms, but the architectural design of SNNs is different. These two paradigms involve the same phase in the framework, and only oneparadigm is chosen for concept integration, depending on the assumption that whether multiple sensory input is independent before integration.

**Figure 2:**
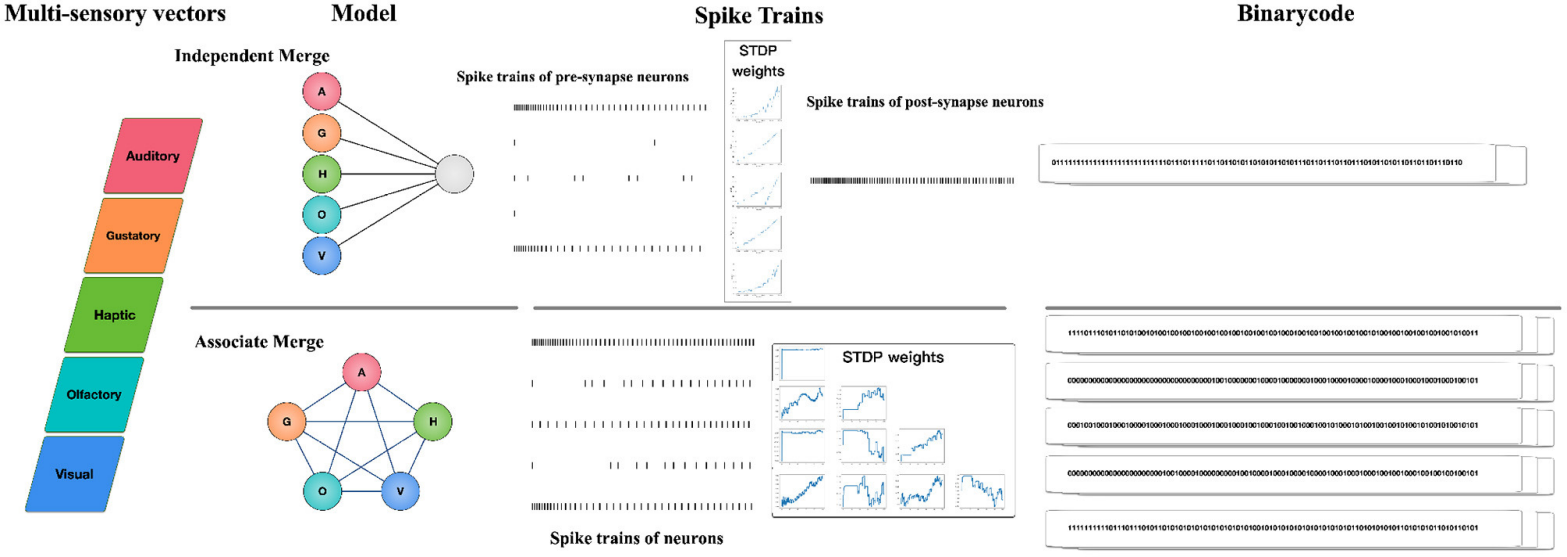
The framework of multisensory concept learning framework based on spiking neural networks. Firstly, for each modality of the concept, we employ a neural model and transform its perceptual strength in the concept's multisensory vector into external stimuli to the neuron; Secondly, the architecture of SNN is designed according to different assumptions. We choose the IM paradigm if we assume that multiple senses are independent of each other before fusion, and we choose the AM paradigm if we assume that multiple senses are associated with each other; Thirdly, we specify the neuron model in SNN and sequentially feed concepts to the network, with STDP rules adjusting the network's connection weights. Given the running interval [0, *T*], we record the spike trains of each neuron; Finally, we convert the spike trains of specific neurons into binarycode as the final integrated representation.

Figure 2 illustrates the workflow: Firstly, for each modality of the concept, we employ a neural model and transform its perceptual strength in the concept's multisensory vector into external stimuli to the neuron (we work on five sensory modalities: auditory, gustatory, haptic, olfactory, visual, so the dimensions of the multisensory vector is five); Secondly, the architecture of SNN is designed according to different assumptions. We choose the IM paradigm if we assume that multiple senses are independent of each other before fusion, and we choose the AM paradigm if we assume that multiple senses are associated with each other; Thirdly, we specify the neuron model in SNN and sequentially feed concepts to the network, with STDP rules adjusting the network's connection weights. Given the running interval [0, *T*], we record the spike trains of each neuron; Finally, we convert the spike trains of specific neurons into binarycode as the final integrated representation. The framework is described in detail with the IM and AM paradigms individually in the following sections.

### 3.1. The Framework

#### 3.1.1. Independent Merge

The IM paradigm is founded on the commonly used cognitive psychology assumption that information for each modality of the concept is independent before integration. It's a two-layer spiking neural network model, with five neurons corresponding to the stimuli of the concept's five separate modal information in the second layer, and a neuron reflecting the neural state after multisensory integration in the second layer. We record the spiking train of the postsynaptic neuron and transform them into integrated vectors for the concept.

For each concept, we get its representation from human-labeled vectors, $\vec{m}=\left[m_{A},m_{G},m_{H},m_{O},m_{V}\right]$. The subscripts here represent the concept's perceptual strength as indicated by auditory, gustatory, haptic, olfactory, and visual senses. We min-max normalize the multisensory representation of the concept in the dataset as input to the model during the data preparation stage such that each value of the vector is between 0 and 1. In LC823, for instance, the vector for the concept “honey” is [0.13, 0.95, 0.57, 0.75, 0.80]. We employ LIF or Izhikevich as presynaptic neural models and IF as postsynaptic neural models independently for the generality of the framework. Initially, for each presynaptic neuron, we regard the current *I* = *m*_i_*I_*boost*_ as the stimuli to the neuron *where i* ∈ [*A, G, H, O, V*] The the conductance *g*_*e*_ of the postsynaptic neuron is updated whenever the presynaptic neuron fires as *g*_*e*_←*g*_*e*_+Δ*W*_*ij*_, and the postsynaptic neuron generates spikes based on the IF model. The synaptic strength between the postsynaptic neuron and the presynaptic neuron is referred to as the weight Δ*W*_*ij*_ in this case. The initial weights between presynaptic and postsynaptic neurons $W_{0}^{i}=\frac{g_{i}}{\Sigma_{i}^{n}g_{i}}$ where $g_{i}=\frac{1}{\sigma_{i}^{2}}$,$\sigma_{i}^{2}$ represents the variance for each kind of multisensory data. They are calculated using the Bayesian formula and the assumption that each modal is independent before to fusion (details in the Appendix). At the same time, the spike trains of presynaptic and postsynaptic neurons will dynamically adjust to the weights in accordance with the STDP law. During [0, *T*], we record the spike train of the postsynaptic neuron *S*^*post*^([0, *T*]) and transform them into binarycode *B*^*post*^([0, *T*]), as the final integration representation for the concept in the following manner:


(8) $$\begin{array}{l} \;B^{post}([0,T])=[T(S^{post}((0,tol])),T(S^{post}((tol,2*tol])),\cdots ,\\ T(S^{post}(((k-1)*tol,k*tol])),\cdots ,T(S^{post}((\left\lfloor \frac{T}{tol}\right\rfloor *tol,T]))] \end{array}$$

Here T(interval) operation means that if there is any spikes in the interval, then the bit is 1, otherwise it is 0.

#### 3.1.2. Associate Merge

The AM paradigm assumes that the information for each modality of the concept is associate before integration. It's a five-neuron spiking neural network model, with five neurons corresponding to the stimuli of the concept's five separate modal information. They are connected to one another, and there are no self-connections. We record the spiking trains of all neurons and transform them into integrated vectors for the concept.

We use LIF or Izhikevich neural models to model each neuron for the generality of the framework. For each concept, we get its normalized representation from human-labeled vectors, $\vec{m}=\left[m_{A},m_{G},m_{H},m_{O},m_{V}\right]$. Initially, for each neuron *i* ∈ [*A, G, H, O, V*], we consider *I* = *m*_i_*I_*boost*_ as the stimuli. The the current *I* of the postsynaptic neuron is updated whenever the presynaptic neuron fires as *I* ← *I* + Δ*W*_*ij*_. And the postsynaptic neuron generates spikes based on the its model. The weight *W*_*ij*_ is the synaptic strength between the presynaptic neuron and the postsynaptic neuron. The initial value for the weight is determined by the correlation each modality pair overall the representation dataset, i.e., *W*_0_ = *Corr*(*i, j*) where *i, j* ∈ [*A, G, H, O, V*], which is different from AM paradigm. Simultaneously, presynaptic and postsynaptic neurons' spike trains will dynamically change to the weights in accordance with the STDP law. We denote *S*^*i*^([0, *T*]) as the *ith* neuron's spike trains during [0, *T*] and corresponding binary vector *B*^*i*^([0, *T*]). And we record the spike trains of all neurons, transform them into binarycode *B*^*i*^([0, *T*]) and concatenate them as the final integration vector *B*([0, *T*]) in the following way:


(9) $$\begin{array}{l} B^{i}([0,T])=[T(S^{i}((0,tol])),T(S^{i}((tol,2*tol])),\cdots ,\\ \;T(S^{i}(((k-1)*tol,k*tol])),\cdots ,T(S^{i}((\left\lfloor \frac{T}{tol}\right\rfloor \\ \;*tol,T]))] \end{array}$$

(10) $$\begin{array}{l} B\left(\left[0,T\right]\right)=[B^{A}(\left[0,T\right])⊕B^{H}\left(\left[0,T\right]\right)⊕B^{G}\left(\left[0,T\right]\right)⊕B^{O}\left(\left[0,T\right]\right)\\ \;⊕B^{V}(\left[0,T\right])] \end{array}$$

## 4. Experiments

### 4.1. Concept Similarity Test

Concept similarity test is commonly used in the field of artificial intelligence to evaluate the effectiveness of system-generated representations (Agirre et al., 2009). Generally, humans score the similarity of a particular concept pair, while the concept pair corresponds to the system-generated representation to calculate the similarity score. After the two scores are ranked in the measure dataset, the Spearman's correlation coefficient is calculated to reflect how close the system-generated representations are to humans. In this article, we evaluate the closeness of the concepts' original or multisensory integration representations and human beings with WordSim353 (Agirre et al., 2009) and SCWS1994 (Huang et al., 2012).

#### 4.1.1. The Experiment

To thoroughly test our framework, we did experiments for IM and AM paradigms with three multisensory datasets (BBSR, LC823, Lancaster40k) respectively and analyzed the effectiveness differences between the representations after SNN integration and the original representations. In the experiments, both IM and AM paradigms involve a unique parameter in the process of conversion from spike trains to binarycode: the tolerance *tol*. It represents the size of the reducing window for converting spike trains in the time interval into binarycode, which reflects the strength of compressing the spike sequence into a integrated binarycode. In each dimension of the integrated vector, a larger *tol* signifies a higher degree of information compression and a bigger reducing window, and *vice versa*. But, if *tol* is too small, the representation vector's dimensionality will be too large, and if *tol* is too big, the diversity of all representations will be damaged. Therefore, we traverse *tol* across the range [0, 500] while restricting diversity to the range [0.05, 0.95], and the results indicate the present model's ideal results as well as the matching *tol*.

We used the evaluation datasets WordSim353 and SCWS1994 for testing, and the inputs of the models were from different sources of multisensory representation datasets: BBSR, LC823an, Lancaster40k, and tested using two paradigms, IM and AM, respectively. For the AM paradigm, Izhikevich's seven models and LIF model were used, while for the IM paradigm, IF model were used for postsynaptic neurons and Izhikevich's seven models and LIF model were used for presynaptic neurons. The running time of all the tests is 100 ms and *I*_*boost*_ = 100.

#### 4.1.2. Results and Analysis

From the overall results for both IM and AM paradigms, the integrated vectors are closer to humans than the original vectors based on our models: 37 submodels achieved better results for a total of 48 tests for both IM and AM, as Table 2 shows. In terms of overall dataset, 15/16 tests work better for the BBSR dataset, 14/16 tests work better for LC823an, and 8/16 tests work better for Lancaster40k.

**Table 2:** Concept similarity test results.

**Merge way**	**Model**	**BBSR**				**LC823an**				**Lancaster40k**			
		**Tol**	**WordSim353**	**SCWS1994**	**Average**	**Tol**	**WordSim353**	**SCWS1994**	**Average**	**Tol**	**WordSim353**	**SCWS1994**	**Average**
Origin	–	–	0.4182	0.5838	0.5010	–	0.1321	0.5525	0.3423	–	0.2640	**0.3974**	0.3534
AM	Izh-RZ	93	0.3455	0.6089	0.4772	165	0.3804	0.4260	0.4032	9	0.3560	0.3295	0.3427
	Izh-FS	95	0.4955	0.5659	0.5307	312	0.4223	0.3788	0.4006	9	0.3787	0.3471	0.3629
	Izh-IB	384	0.5455	0.5870	0.5662	32	0.3696	0.5277	0.4486	25	0.3388	0.3818	0.3603
	Izh-LTS	174	0.5068	0.6127	0.5598	17	0.3107	0.5390	0.4249	16	0.3557	0.3629	0.3593
	Izh-RS	366	0.4955	0.5857	0.5406	84	0.5179	0.5271	0.5225	55	0.3206	0.3708	0.3457
	Izh-CH	170	0.4273	0.5928	0.5100	7	0.1089	0.4884	0.2986	14	0.3150	0.3349	0.3249
	Izh-TC	148	0.5068	0.6103	0.5586	6	0.2214	0.5181	0.3698	7	**0.3979**	0.3364	0.3672
	LIF	187	0.5727	0.6927	0.6327	330	0.5036	**0.6330**	0.5683	86	0.1788	0.3500	0.2644
IM	Izh-RZ	17	0.4636	0.634	0.5488	10	0.5545	0.5618	0.5581	4	0.2026	0.3139	0.2583
	Izh-FS	17	0.4636	0.6388	0.5512	10	0.5545	0.5617	0.5581	21	0.3371	0.2910	0.3140
	Izh-IB	7	**0.5477**	0.5988	**0.5733**	24	0.5509	0.5491	0.5500	31	0.1597	0.3040	0.2319
	Izh-LTS	83	0.5000	**0.6417**	0.5708	18	**0.6080**	0.5361	**0.5721**	56	0.3610	0.3448	0.3529
	Izh-RS	196	0.5023	0.5530	0.5276	163	0.4830	0.4613	0.4722	68	0.0757	0.2959	0.1858
	Izh-CH	94	0.4659	0.5786	0.5222	8	0.5696	0.4746	0.5221	50	0.3843	0.3813	**0.3828**
	Izh-TC	17	0.4636	0.6125	0.5381	5	0.4509	0.5310	0.4909	20	0.3387	0.3042	0.3215
	LIF	143	0.4205	0.6167	0.5186	3	0.0643	0.5672	0.3158	324	0.0018	0.1481	0.1965

*The bold values indicates the current measure dataset reflect the best results, while the underlined values imply that the multisensory integrated representation is closer to humans than the original representation*.

In almost all experiments, multisensory integrated representations based on our framework outperform unintegrated ones, with the exception of the instability shown in IM and AM paradigms when Lancaster40k is employed as the input. For any of the multisensory vectors, an integration way could be found to improve their representations.

### 4.2. Comparisons Between IM and AM Paradigms

Unlike the analysis of the macro-level above, in this section we introduce the concept feature norms to compare IM and AM paradigms from the micro-level perspective of each concept. Concept feature norms are a way of representing concepts by using standardized and systematic feature descriptions that mirror human comprehension. The similarities and differences of concepts are related to the intersection and difference of concept feature norms. McRae's concept feature norms, introduced by McRae et al. (2005), are the most prominent work in this area. They not only supplied 541 concepts with feature norms, but also proposed a methodology for generating them. For example, the feature norms of the concept “basement” are “*used for storage,” “found below ground,” “is cold,” “found on the bottom floor,” “is dark,” “is damp,” “made of cement,” “part of a house,” “has windows,” “has a furnace,” “has a foundation,” “has stairways,” “has walls,” “is musty,” “is scary,” and “is the lowest floor.”* Another semantic feature norms dataset analogous to McRae is CSLB (Centre for Speech, Language, and the Brain). They collected 866 concepts and improved the feature normalization and feature filtering procedure (Devereux et al., 2014). The McRae and CSLB criteria for human conceptual cognition are used in this research to investigate how each concept is similar to human cognition.

We compare and analyze IM and AM paradigms from two perspectives. First, we use the perceptual strength-related metric Modality Exclusivity to compare the two paradigms to explore the sensitive of them to the concepts' strength distribution of multisensory information. Then, to assess the generality of the IM and AM paradigms, we introduce nine psycholinguistic dimensions derived from the concept's nature, which are unrelated to perceptual strength.

#### 4.2.1. Modality Exclusivity

**Figure 3:**
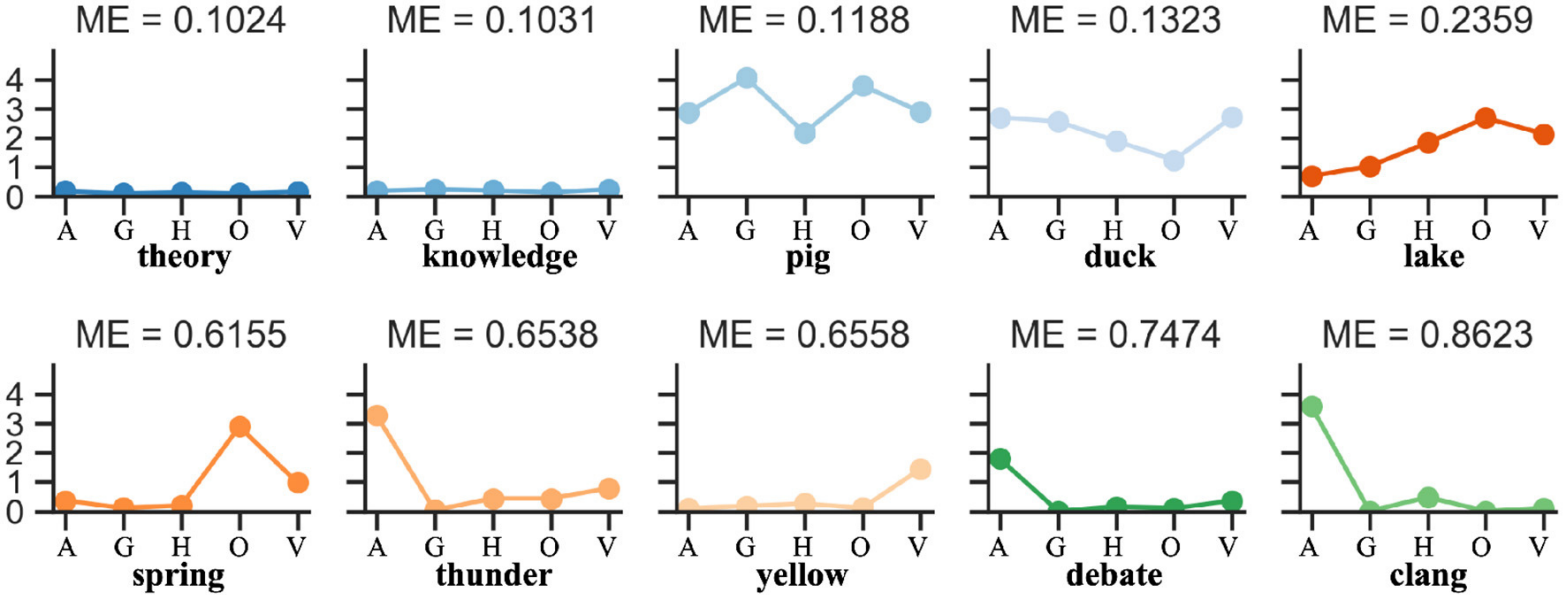
Modality exclusivity demonstration. Modality exclusivity (ME) is a metric measuring how much of a concept is perceived through a single perceptual modality. For each concept, the value of ME is calculated as the perceptual strength range divided by the sum, and spanning from 0 to 100% for completely multimodal to completely unimodal perception.

Modality Exclusivity (ME) is a metric measuring how much of a concept is perceived through a single perceptual modality (Lynott and Connell, 2013). For each concept, the value of ME is calculated as the perceptual strength range divided by the sum, and spanning from 0 to 100% for completely multimodal to completely unimodal perception. Figure 3 show some examples.

In the concept feature norms dataset, we first obtain all similar concepts *c*^*similar*^ for each concept *c* based on the number of feature overlaps and record their rank list $R_{c}^{similar}$ sorted by similarity. Then, for all concepts, the corresponding multisensory integrated binary representations *B*^*IM*^ and *B*^*AM*^ are produced using the IM and AM paradigms, respectively. Next, for concept *c*, its *k* similar concepts $c_{IM}^{k\;similar}$ and $c_{AM}^{k\;similar}$ are computed based on integrated binarycodes and harming distance, respectively. We query the rank of these *k* similar concepts in the feature norms space $R_{c}^{similar}$ and take the average value, denoted as *kAR*_*c*_*IM*__ and *kAR*_*c*_*AM*__, which reflects the closeness of the multisensory representations to human cognition using two ways of integration in our framework. Smaller values of *kAR* indicate closer to human cognition at the microscopic level. Finally, we focus on all concepts in the representation dataset and calculate the correlation coefficients between the *kAR*_*c*_*IM*__ or *kAR*_*c*_*AM*__ arrays obtained using the above approach and the ME arrays corresponding to the concepts. This coefficient reflects the correlation between the two different multisensory concept integration paradigms and modal exclusivity. And in this experiment we only test the Izhikevich model and set k to 5.

**Figure 4:**
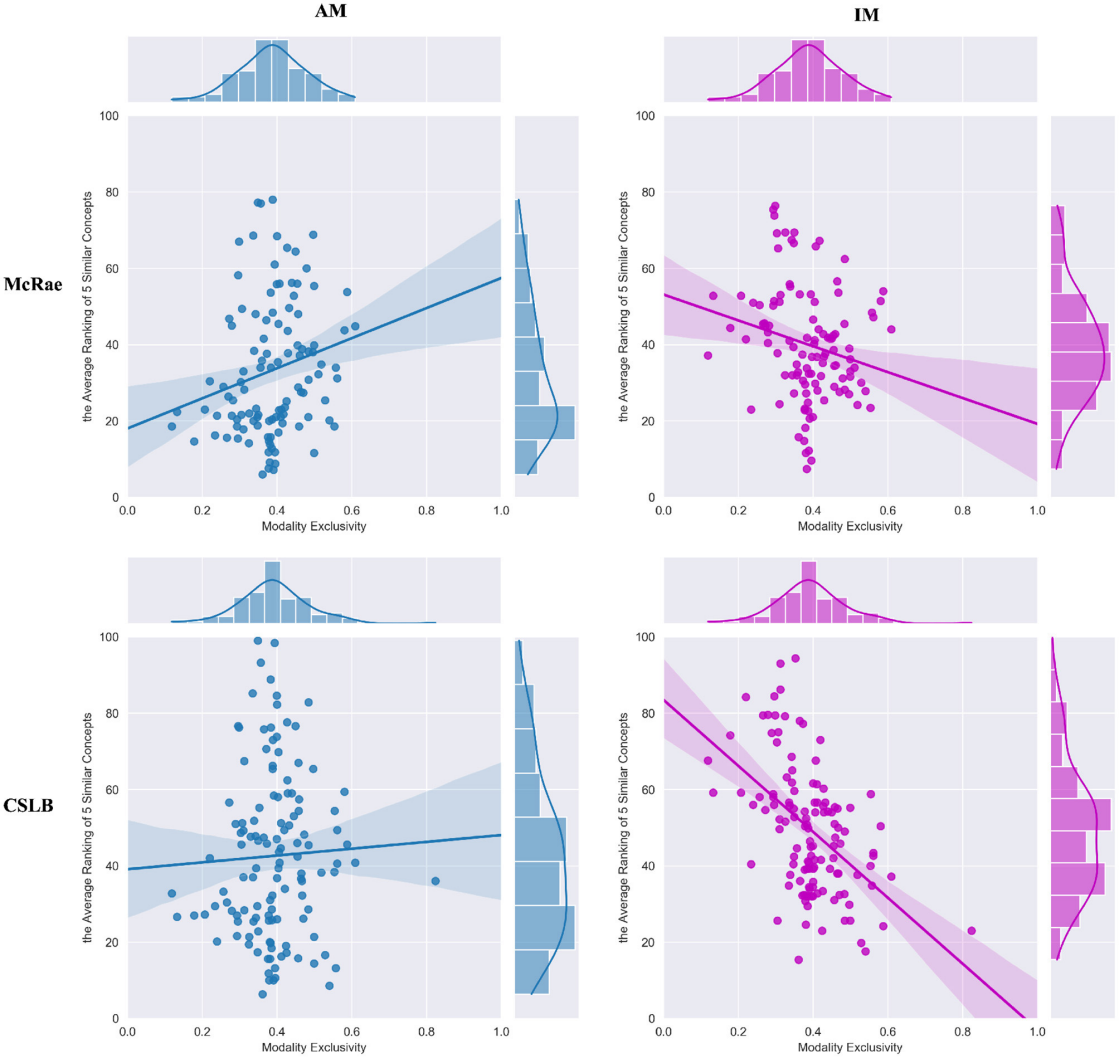
The correlation between ME and average of five similar concept rankings.

The results in Table 3 reveal the difference between IM and AM paradigms. The IM paradigm has a stronger negative correlation in both concept feature norms test sets, but the AM paradigms has a slightly positive correlation. We investigate this discrepancy further by viewing the FS model in detail, as shown in Figure 4. The results reveal that for concepts with higher ME (such as “spring,” “thunder,” “yellow,” “debate,” “clang” in Figure 3), the IM paradigm is better at multisensory integration. While the AM paradigm is less input biased for each modality, it benefits the concept of uniform modal distribution (such as “theory,” “knowledge,” “pig,” “duck,” “lake” in Figure 3).

**Table 3:** The sensibility of IM and AM results to modality exclusivity.

**Izhkevich model**	**AM**		**IM**	
	**McRae**	**CSLB**	**McRae**	**CSLB**
RZ	0.0149	−0.0987	−0.1524	−0.4848
FS	0.2679	0.0901	−0.134	−0.4447
IB	−0.0559	0.0191	−0.2672	−0.4986
LTS	0.2113	0.035	−0.12	−0.0453
RS	0.1943	−0.0087	−0.006	−0.1997
CH	0.0988	0.0197	0.0294	0.0964
TC	0.2078	0.0398	−0.2115	−0.4761

#### 4.2.2. Generality Analysis

The ME metric used in the previous experiments is a perceptual strength-related indicator for the concept representation. In this part, we will test the framework from the input concept itself. And we introduce Glasgow norms which are a set of normative assessments on nine psycholinguistic dimensions: arousal (AROU), valence (VAL), dominance (DOM), concreteness (CNC), imageability (IMAG), familiarity (FAM), age of acquisition (AOA), semantic size (SIZE), and gender association (GEND) for 5,553 concepts (Scott et al., 2019).

In the same manner as the previous experiment. In concept feature norms, we first record all similar concepts for each concept, then sort them by similarity and rank them. Then, for IM and AM paradigms, we use the same concept input, get the integration vector for each concept, find their k similar, and get the mean value of their ranking in concept feature norms as *kAR*_*c*_*IM*__ and *kAR*_*c*_*AM*__. Finally, we determine the correlation coefficient between each psychological characteristic and the concept's average ranking value *kAR* for the two paradigms. We still only test the Izhikevich model in this experiment, and the value is set to 5.

**Figure 5:**
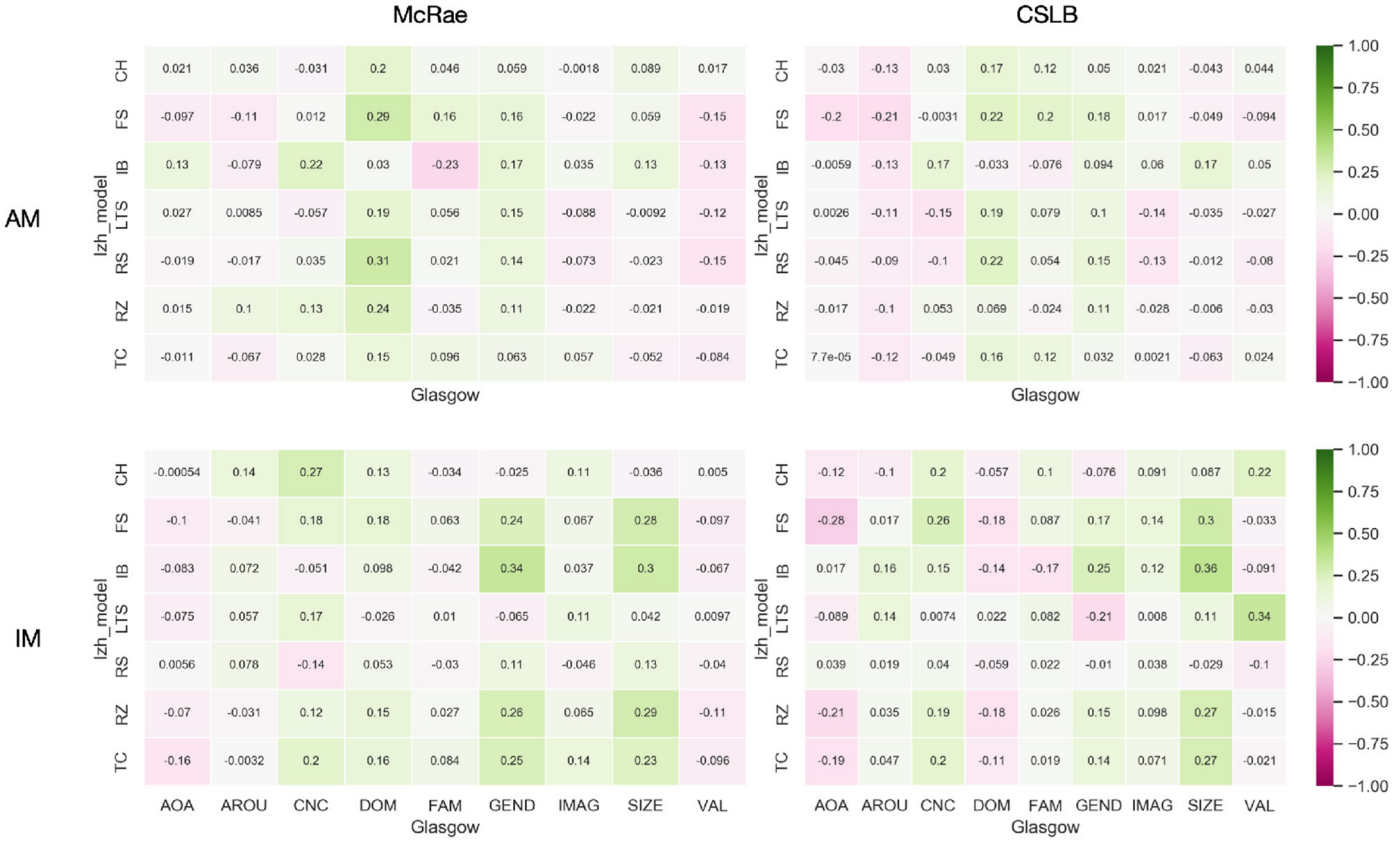
The heatmap of generality analysis results.

**Figure 6:**
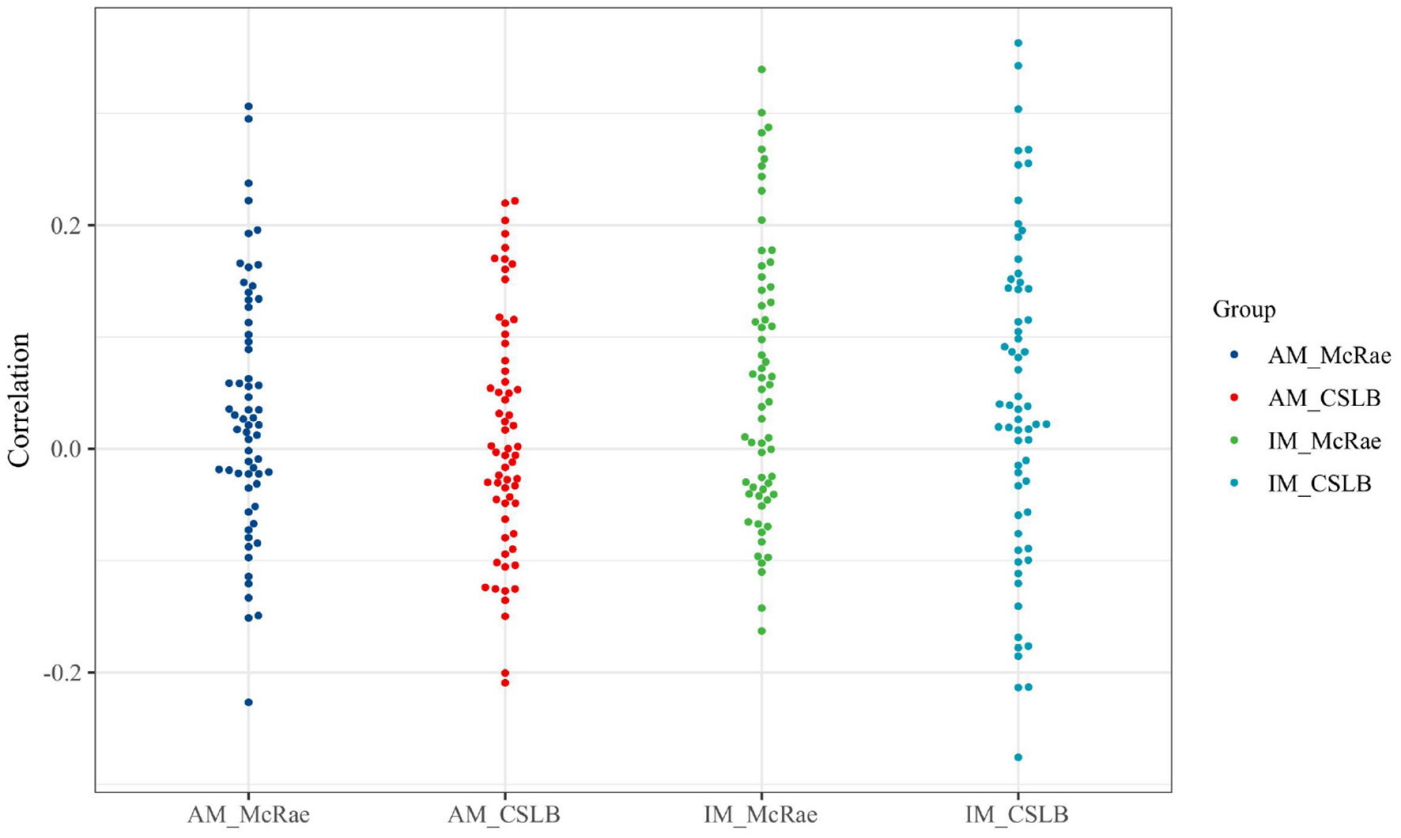
The beeswarm of correlation distribution.

We used heatmaps (Figure 5) to visualize the correlation coefficients between the IM and AM paradigms' *kAR* and nine psycholinguistics in the two concept feature norms sets McRae and CSLB. Additionally, we omit the adopted Izhikevich submodels and provide the correlation coefficients using a beeswarm (Figure 6) to explain them more clearly.

According to the experimental results presented, the absolute values of all correlation coefficients are <0.3. The effect of vectors after integration of either IM or AM paradigms does not have any relationship with the nature of the concepts for several dimensions, including AOA, AROU, FAM, IMAG, and VAL. This indicates that both paradigms have good generality and the framework is not affected by the concepts themselves.

## 5. Discussion

In this study, we propose a SNN-based concept learning framework for multisensory integration that can generate integration vectors based on psychologist-labeled multimodal representations. Vision, hearing, touch, smell, and taste are among the five modalities used in our research, which also includes a brain-like SNN model. We intend to add more brain-like processes in the future, such as multisensory fusion plasticity. The multisensory data we currently use are labeled by cognitive psychologists, which is relatively expensive and small, and in the future we consider expanding the relevant dataset by mapping for larger scale experiments. The current research focuses on multisensory representation of concepts, which is a subset of pattern representation in AI, and future research can be deeply integrated with downstream tasks to create AI systems that incorporate multisensory integration. At the same time, this places more demands on multisensory perceptrons. Human perception of concepts has not only multisensory perception but also more textual information based on abstract information, and it is also worth exploring how to combine these two parts to build human-like concept learning systems in the future.

## Data Availability Statement

Publicly available datasets were analyzed in this study. This data can be found at: http://osf.io/7emr6/; http://www.neuro.mcw.edu/resources.html; https://link.springer.com/article/10.3758/BRM.41.2.558; https://link.springer.com/article/10.3758/s13428-012-0267-0.

## Author Contributions

YW and YZ designed the study, performed the experiments, and wrote the manuscript. Both authors contributed to the article and approved the submitted version.

## Funding

This study was supported by the Strategic Priority Research Program of the Chinese Academy of Sciences (Grant No. XDB32070100).

## Conflict of Interest

The authors declare that the research was conducted in the absence of any commercial or financial relationships that could be construed as a potential conflict of interest.

## Publisher's Note

All claims expressed in this article are solely those of the authors and do not necessarily represent those of their affiliated organizations, or those of the publisher, the editors and the reviewers. Any product that may be evaluated in this article, or claim that may be made by its manufacturer, is not guaranteed or endorsed by the publisher.

## Appendix

### The Initial Weights in IM

Similar to what cognitive psychologists (Ursino et al., 2014) have done before, we assume that for the concept *s* and its each modality *i* ∈ [*A, G, H, O, V*] representations, *p*(*x*_*i*_|*s*) ~ *N*(*x*_*i*_; *s*, σ_*i*_), where *N*(*x*; μ, σ) stands for the normal distribution over *x* with mean μ and standard deviation σ. They are conditionally independent from each other and by Bayes' rule,

(11)
$$\begin{array}{l} p\left(s|x_{A},x_{G},x_{H},x_{O},x_{V}\right)\propto p\left(x_{A},x_{G},x_{H},x_{O},x_{V}|s\right)\\ \;\propto \underset{i}{\prod }p\left(x_{i}|s\right)=\frac{1}{\prod_{i}\left(\sqrt{2\pi }\sigma_{i}\right)}e^{-\Sigma_{i}\frac{{\left(x_{i}-s\right)}^{2}}{2\sigma_{i}^{2}}}\\ \;\propto -\Sigma_{i}\frac{{\left(x_{i}-s\right)}^{2}}{2\sigma_{i}^{2}} \end{array}$$

The maximum-a-posteriori estimation for *s* is $\hat{s}=\Sigma_{i}\frac{\frac{1}{\sigma_{i}^{2}}}{\Sigma_{i}\frac{1}{\sigma_{i}^{2}}}x_{i}$, where $\frac{1}{\sigma_{i}^{2}}$ reflects the reliability of each modality for the same concept *s*. In our IM schema, we regard normalized reliability as the initial weights between pre-synaptic neurons (describing each modality) and the post-synaptic neuron(for integration), i.e.,

(12)
$$\begin{array}{l} w_{i}^{0}=\frac{\frac{1}{\sigma_{i}^{2}}}{\Sigma_{i}\frac{1}{\sigma_{i}^{2}}} \end{array}$$

where we can get each σ_*i*_ *via* psychologist-labeled multisensory datasets.
